# Blood lipids, homocysteine, stress factors, and vitamins in clinically stable multiple sclerosis patients

**DOI:** 10.1186/1476-511X-9-19

**Published:** 2010-02-18

**Authors:** Giuseppe Salemi, Maria Concetta Gueli, Francesco Vitale, Floriana Battaglieri, Egidio Guglielmini, Paolo Ragonese, Angela Trentacosti, Maria Fatima Massenti, Giovanni Savettieri, Antonino Bono

**Affiliations:** 1 Department of Clinical Neuroscience, University of Palermo, Palermo, Italy; 2 Department of Biochemical Sciences, University of Palermo, Palermo, Italy; 3 Department of Sciences for Health Promotion - Hygiene section, University of Palermo, Palermo, Italy; 4 Department of Medical Biotechnologies and Forensic Medicine, University of Palermo, Palermo, Italy

## Abstract

Multiple Sclerosis (MS) patients present a decrease of antioxidants and neuroprotective and immunoregulatory vitamins and an increase of total homocysteine (tHcy), cholesterol (CHL), HDL-cholesterol, and of cellular stress markers, variably associated with the different phases of the disease. We compared the blood levels of uric acid, folic acid, vitamins B12, A, and E, tHcy, CHL, HDL-cholesterol, and triglycerides in forty MS patients during a phase of clinical inactivity with those of eighty healthy controls, matched for age and sex. We found higher levels of tHcy (p = 0.032) and of HDL-cholesterol (p = 0.001) and lower levels of vitamin E (p = 0.001) and the ratio vitamin E/CHL (p = 0.001) in MS patients. In conclusion, modifications of some biochemical markers of cell damage were detected in MS patients during a phase of clinical inactivity.

## Findings

A decrease of antioxidants [1], and neuroprotective [2,3] and immunoregulatory vitamins [3,4] and an increase of total homocysteine (t-Hcy) [5], cholesterol (CHL), HDL-cholesterol [6], and of cellular stress markers [7,8] were reported in patients affected by multiple sclerosis (MS). These changes are regarded as markers of autoimmunity or neurodegeneration. Recently, in view of the unreliability of these results, mainly due to the heterogeneity of the samples investigated, attention has focused on clinical relapse; and active MS has proved to be associated with a decrease of uric acid (UA) and an increase of CHL and stress markers [1,6,8]. To characterize the biochemical status of MS during a phase of clinical inactivity, we compared the blood levels of UA, folic acid (FA), vitamins B12, A, and E, t-Hcy, CHL, HDL-cholesterol, and triglycerides (TG) in stable MS patients with those of healthy controls.

Forty consecutive MS patients with relapsing-remitting or secondary-progressive courses and in a phase of clinical inactivity, participated in the study. They had been free of steroids for at least one month and of immunomodulatory or immunosuppressive drugs for at least two months. The study protocol was approved by the local Ethics Committee. Formal written consent was obtained from each patient. Two venous blood samples were obtained from patient with a time lag of one month in between. A number of healthy individuals were enrolled after their formal written consent. Controls were matched for age (+2 years) and gender to the patients in the ratio of two controls for each case. A venous blood sample was obtained from each control. Pregnant women, habitual smokers, alcohol drinkers, and consumers of vitamins-rich products were excluded. Among the cases, individuals with other disorders in addition to MS were also excluded. Venipuncture, using masked materials, was performed in fasting subjects who on the two previous days had avoided certain habits (alcohol consumption, cigarettes, cigars, and pipe smoking) and foods (red meat, liver, kidney, spleen, fish and sea food, salted food and fat cheese, sweets, grapes, bananas, figs, persimmons, cherry, nuts, and the like) which could have modified the concentration of the molecules to be detected. The Ubbink method was used to establish plasma levels of tHcy [9]. Competitive magnetic separation on a Technicon Immuno autoanalyser was used for serum FA and vitamin B12 assays. High-performance liquid chromatography and fluorimetry were used for vitamin A and vitamin E assays. Commercially available enzymatic colorimetric tests were used for UA, CHL, HDL-cholesterol, and TG assays (Roche Diagnostics GmbH, Mannheim). The vitamin E/CHL ratio was calculated. Mean differences in normally distributed data between cases and controls were analysed by Student's t test. As tHcy, FA, UA, vitamin A, and vitamin E concentrations were not uniformly distributed, median values and the Mann-Whitney U test were used for comparison of the patient and control groups. To increase dosage reliability; patients had two different venipunctures and their mean value was used for calculations.

We included 40 MS patients and 80 healthy controls. The patients' median age was 39.5 years (range 16 - 58); twelve were male; mean MS duration was 11.95 ± 9.28 years; median EDSS was 3.0 (range 1.0 - 6.0); mean MSSS was 3.95 ± 1.95; 28 patients had a relapsing-remitting course and 12 had a secondary-progressive course. Because of the matching criteria, the control group had the same distribution for age (median age: 38.5 years; range 16 - 56) and gender. Table 1 shows the results obtained for each molecule comparing the cases and the control groups. The concentration of UA, FA, TG, and vitamin A was similar between patients and controls. Vitamin B12 and CHL concentration was higher in MS patients, but the difference between the two groups did not reach statistical significance. The concentration of HDL-cholesterol and Hcy was higher in the MS group, while the concentration of vitamin E and the ratio of vitamin E and CHL (E/CHL) were lower in the MS group; these differences were statistically significant.

**Table 1:** Comparison of Blood Lipids, total Homocysteine, Uric Acid and Vitamins concentration in subjects with a Multiple Sclerosis and in healthy controls.

Molecule^a^	Patients	Controls	P°
Vitamin B12	666.42 ± 354.16	493.70 ± 229.16	0.058
Folic acid	7.3 (2.8, 25.0)	5.9 (1.2, 16.0)	0.196
Uric acid	4.3 (2.8, 5.9)	4.1 (2.1, 7.3)	1.000
Homocysteine	9.3 (4.0, 86.5)	6.3 (3.2, 15.0)	0.032
Vitamin A	0.3 (0.1, 0.7)	0.4 (0.1, 2.4)	0.149
Vitamin E	12.0 (5.4, 26.5)	22.5 (1.9, 70.2)	0.001
Cholesterol	187.70 ± 40.30	169.07 ± 35.18	0.071
HDL-cholesterol	61.42 ± 14.85	50.05 ± 10.81	0.001
Triglyceride	92.82 ± 46.72	82.35 ± 44.52	0.401
E/CHL	0.07 ± 0.04	0.15 ± 0.08	0.001

*Vitamin B12 (pg/ml), total Cholesterol (mg%), HDL-cholesterol (mg%) and Triglyceride (mg %) had a normal distribution; mean values and standard deviation are reported. Folic acid (ng/ml), Uric acid (mg %), total Homocysteine (μmol/L), vitamin A (mgr/L), and vitamin E (mgr/L) had not a normal distribution; median values, minimum and maximum are reported.

° Student "t test" was used to calculate statistical significativity for vitamin B12, cholesterol, HDL-cholesterol, triglycerid, and E/CHL ratio. Mann-Whitney U test was used to calculate statistical significativity for folic acid, uric acid, homocysteine, vitamin A, and vitamin E.

This study shows that the MS patients in a phase of clinical inactivity presented modifications of some biochemical markers of cell damage. The study's main weakness is its limited sample size and it must therefore be regarded as preliminary to larger investigations. However, the study also has some strong points: it restricted inclusion to patients in a clinically stable phase, it excluded primary progressive MS patients, and on the days preceding the venipuncture it excluded the intake of foods or substances that might have modified the concentration of the molecules studied. We found that the MS patients in a phase of clinical stability had higher blood levels of tHcy and HDL-cholesterol and lower blood levels of vitamin E and the E/CHL ratio. The increased levels of tHcy, that we found in our patients, were consistent with those of previous studies performed in MS patients outside relapse [5]. The meaning of the increase, which damaged the folding of proteins by increasing the cleavage of disulphide bridges and was targeted to generate free radicals, is uncertain and requires specific study. Our data agree with those reported elsewhere that tHcy increase was paralleled not by a decrease of vitamin B12 and FA, as usually reported for other disorders, but by normal or increased values of both vitamins [5]. This observation also requires "ad hoc" studies. The increased concentration of HDL-cholesterol in a stable phase of the disease could counteract the increased concentration of cholesterol that occurs in the course of a relapse and ensure that it reaches the liver [10]. Increased values of total and HDL - cholesterol were reported in a study aimed at assessing the correlation between blood CHL and MRI activity after a clinical episode suggesting MS [6]. The decrease of the concentration of vitamin E, the major hydrophobic chain-breaking antioxidant, confirms the possible involvement of this vitamin in MS pathology: vitamin E levels are decreased in the demyelination plaques of MS brains, and vitamin E levels and E/CHL ratios are lower in the serum of MS patients [2]. However, serum vitamin E is not a marker of clinical activity because it is not paralleled by a similar low concentration in the CSF or by a reduction of the [vitamin E_CSF_]/[vitamin E_serum_] ratio. Our data could indicate that vitamin E was consumed to counterbalance MS chronic neurodegeneration.

We observed no differences as regards UA, FA, vitamin A, vitamin B12, CHL, and TG between MS patients in a phase of clinical inactivity and healthy controls, which means that they are unlikely to be involved in the biochemical processes taking place during this phase of MS. However, the role of UA concentration in MS outside a clinical relapse remains controversial. Some researchers did not find any association with MS, agreeing with our results; others found an increase of UA both in serum and in cerebrospinal fluid (CSF) [1,7,11]. These conflicting results could be explained by differences in the populations studied or by different detection methods and they require further investigation for a better understanding of the role of UA in the biochemical changes occurring in the course of MS.

## Competing interests

The authors declare that they have no competing interests.

## Authors' contributions

GSal and MCG conceived the study and interpreted the data. FV, GSav, and AB were responsible for critical revision and supervision. FB and AT contributed to data collection and the critical revision of the manuscript. EG was responsible for the Hcy and vitamin analyses and for interpretation of data and critical revision of the manuscript. MFM was responsible for the fatty acid and UA analyses and interpretation of data and critical revision of the manuscript. PR was responsible for data collection, handling of the database, statistical analyses, and critical revision of the manuscript. All of the authors have read and approved the final version of the manuscript.
